# Siamese comparative transformer-based network for unsupervised landmark detection

**DOI:** 10.1371/journal.pone.0313518

**Published:** 2024-12-31

**Authors:** Can Zhao, Tao Wu, Jianlin Zhang, Zhiyong Xu, Meihui Li, Dongxu Liu

**Affiliations:** 1 National Key Laboratory of Optical Field Manipulation Science and Technology, Chinese Academy of Sciences, Chengdu, China; 2 Key Laboratory of Optical Engineering, Chinese Academy of Sciences, Chengdu, China; 3 Institute of Optics and Electronics, Chinese Academy of Sciences, Chengdu, China; 4 University of the Chinese Academy of Science, Beijing, China; Purdue University, UNITED STATES OF AMERICA

## Abstract

Landmark detection is a common task that benefits downstream computer vision tasks. Current landmark detection algorithms often train a sophisticated image pose encoder by reconstructing the source image to identify landmarks. Although a well-trained encoder can effectively capture landmark information through image reconstruction, it overlooks the semantic relationships between landmarks. This contradicts the goal of achieving semantic representations in landmark detection tasks. To address these challenges, we introduce a novel Siamese comparative transformer-based network that strengthens the semantic connections among detected landmarks. Specifically, the connection between landmarks with the same semantics has been enhanced by employing a Siamese contrastive regularizer. In addition, we integrate a lightweight direction-guided Transformer into the image pose encoder to perceive global feature relationships, thereby improving the representation and encoding of landmarks. Experiments on the CelebA, AFLW, and Cat Heads benchmarks demonstrate that our proposed method achieves competitive performance compared to existing unsupervised methods and even supervised methods.

## 1 Introduction

Landmark detection focuses on capturing specific information that represents the local features of objects within images. This technology has become increasingly prevalent in downstream computer vision tasks, including pose estimation [1, 2], behaviour recognition [3, 4], and motion capture [5, 6]. The majority of prior works [7–10] in landmark detection have employed fully supervised approaches, which encounter a significant challenge because of the high cost associated with obtaining labelled data. As a result, numerous researchers [11–15] have concentrated on advancing unsupervised landmark detection methods that obviate the necessity for manual annotations.

**Fig 1:**
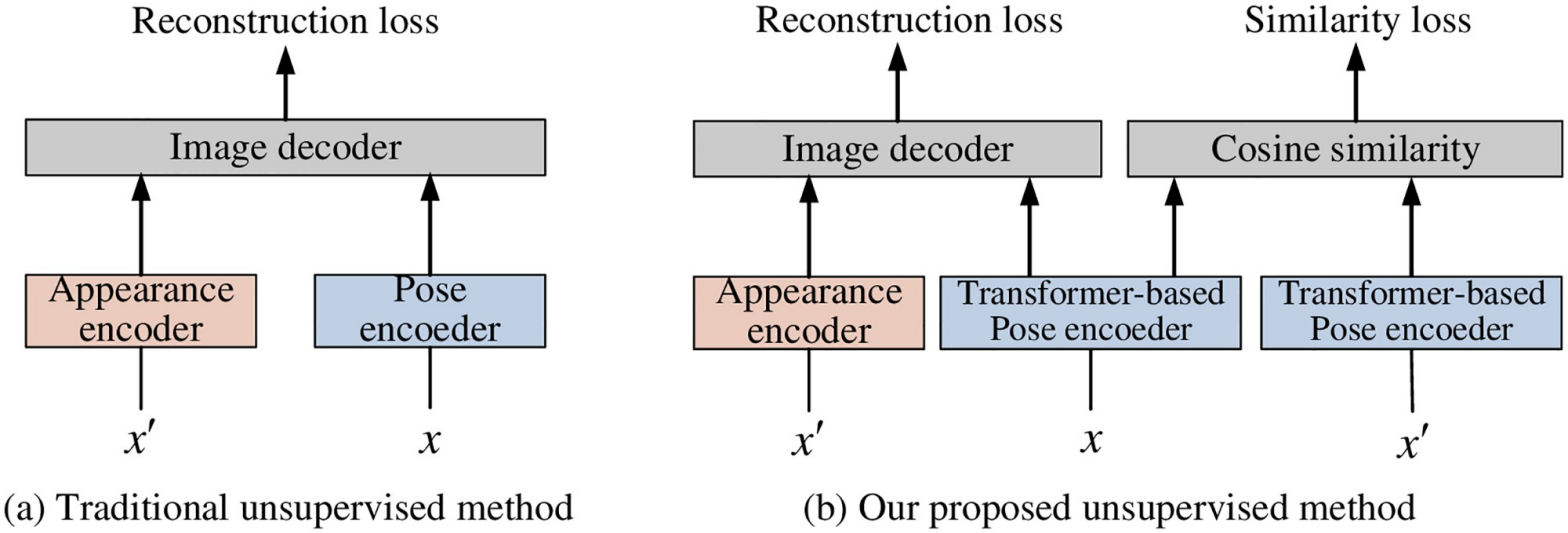
Comparison between (a) traditional unsupervised method and (b) our proposed unsupervised method. We introduce a Transformer-based image pose encoder along with an additional similarity loss to enhance landmark detection capabilities.

Generally, unsupervised learning methods for landmark detection rely on proficiently trained image feature encoders during the reconstruction process. As shown in Fig 1, the traditional unsupervised landmark detection methods typically comprise an image appearance encoder, an image pose encoder, and an image decoder. To begin with, data augmentation generates two images, which are then labelled as the source image and the target image, respectively. These two images have the same appearance features and different landmark information. The image appearance encoder takes the source image as input and generates appearance feature vectors, while the image pose encoder produces landmark features of the objects within the target image. The image decoder reconstructs the source image using outputs from the appearance and image pose encoders. During the reconstruction process, the image pose encoder is trained to identify and locate the landmarks in the image accurately. Finally, the landmarks are extracted by finding the position of the maximum response value in landmark heatmaps generated from the image pose encoder.

Despite recent progress in unsupervised landmark detection, methods based on image reconstruction suffer from several critical drawbacks that limit their effectiveness. One of the primary issues lies in their reliance on a single reconstruction loss throughout the training process. While this loss is crucial for ensuring that the reconstructed image resembles the input, it gradually shifts the model’s focus away from the task of accurate landmark detection. As the network optimizes for reconstruction, the learned representations become increasingly biased toward features that aid in recreating the overall image, rather than identifying distinct and semantically meaningful landmarks. This deviation causes the image encoder to prioritize low-level image features, such as texture and color, at the expense of learning robust landmark-specific features. As a result, the landmarks detected often lack clear semantic connections and do not adequately capture the structure of the represented objects. Moreover, most current approaches [12–15] rely on Convolutional Neural Networks (CNNs) for image pose encoding and landmark prediction. While CNNs are effective at extracting local features, they inherently struggle to capture long-range dependencies due to their limited receptive field. Due to the inability to model these global relationships, the network lacks a deep understanding of how different parts of the object relate to each other, leading to suboptimal landmark predictions. Therefore, the predicted landmarks often fail to form a coherent and meaningful spatial structure, reducing the overall accuracy of the detection.

To overcome these drawbacks, we introduce a novel unsupervised landmark detection approach, referred to as the Siamese Comparative Transformer-based Network (*SCTN*), which deals with semantic inconsistency issues and captures the global feature information. More specifically, a Siamese contrast structure is established to maintain semantic consistency between landmarks. Extracting information from image pairs and comparing their semantic feature representations can effectively improve landmark detection capabilities, and break away from the previous pattern of solitary information extraction. Furthermore, we design a hybrid encoding representation network with a Transformer structure to capture the global interaction among landmarks, moving beyond the exclusive reliance on CNNs for local feature extraction. Meanwhile, the complexity of directly using the Transformer Encoder on a sequence of H × W length will be very high, which will significantly increase the computational complexity of the model. Thus, we employ separate execution and fusion of information from the rows and columns of the feature map to accelerate the algorithm’s execution speed. Extensive experiments have been carried out on challenging landmark detection datasets, and the results demonstrate that the proposed framework significantly outperforms the preceding counterparts.

In summary, our main contributions are highlighted in the following aspects:

- We propose a Siamese contrastive regularizer that directly integrates semantic constraints into the image pose encoder, enhancing its ability to capture meaningful landmark information.
- We integrate a lightweight direction-guided Transformer structure into the image pose encoder, which broadens the encoder’s receptive field, leading to more precise landmark localization.
- We introduce an unsupervised Siamese Comparative Transformer-based Network (*SCTN*) that focuses on identifying meaningful landmarks. The experiments demonstrate that *SCTN* achieves outstanding landmark detection performance on three public benchmarks, namely CelebA, AFLW, and Cat Heads.

## 2 Related works

### 2.1 Unsupervised landmark detection

Previous landmark detection methods [7, 16, 17] usually utilized a supervised approach. These methods trained networks by minimizing the absolute distances between points predicted by the model and those provided by human annotators. Recently, researchers have shifted toward unsupervised landmark detection due to its superior performance compared to supervised methods and its advantage of not requiring labelled data. Thewils et al. [11, 18, 19] first proposed viewing landmark detectors as local image descriptors. They expanded the equivariance constraint to ensure invariance in response to image transformations. Mails et al. [15, 20] presented an approach similar to that of Thewils et al., where object landmarks are depicted as a form of descriptor. Conversely, they implemented a cycle of correspondence recovery through clustering. Subsequently, they utilized these pseudo labels obtained from clustering to train the model, enabling it to learn object landmark descriptors without annotated labels. Wang et al. [21] presented a novel unsupervised 3D face reconstruction framework that leverages multi-view geometric constraints to train accurate facial pose and depth maps. Wan et al. [22] proposed a Reference Heatmap Transformer, which enhances the accuracy of facial landmark detection by incorporating reference heatmap information. This method was particularly effective in handling challenging scenarios such as large pose variations, severe occlusions, and complex lighting conditions in facial images. In particular, Jakab et al. [23] proposed a simple framework for unsupervised landmark detection using a standard encoder-decoder architecture. They implemented a tight bottleneck to disentangle the pose and appearance of the object for reconstructing the source image and thereby learning landmarks during the reconstruction process. The core idea of [23] was also shared by [12–14, 24, 25].

While methods relying on image reconstruction have achieved significant success from various perspectives, they often lack semantic supervision between predicted landmarks. Our algorithm retrieves landmark information through an image reconstruction module and a contrastive semantic supervision module. In addition, we incorporate efficient global attention into the image pose encoder to achieve a comprehensive understanding of the overall landmark distribution.

### 2.2 Contrastive learning

Contrastive learning, as a self-supervised learning method, aims to capture the intrinsic structure and features of data through instance discrimination tasks [26]. This method aims to minimize the distance between various augmented features of the same instance and maximize the features of the distance between different instances. Through this process, it learns a powerful feature representation that effectively distinguishes between individual instances based on their intrinsic characteristics. Most contrastive learning methods adopt Siamese networks [27] to enhance the representation of instances. For instance, BYOL [28] proposed a novel approach where two networks predict each other’s feature representations without the need for negative samples. SwAV [29] facilitated feature learning through a process that involves clustering and then swapped cluster assignments between different views of an image. MoCo [30] introduced a momentum encoder alongside a dynamic queue for negative samples. VedioMoCo [31] enhanced MoCo for unsupervised video representation learning by improving the temporal robustness of the encoder and modeling the temporal decay of the keys. SimCLR [32] simplified the contrastive learning framework through data augmentation and batch learning. Simsiam [33] demonstrated that simple Siamese networks, even without relying on negative sample pairs, large patches, or momentum encoders, are capable of learning meaningful representations.

Inspired by their excellent works, we adopt a Siamese Contrastive structure into the landmark detection task. Our goal is to refine the semantic representation of positive landmarks, guaranteeing that the image pose encoder not only accurately identifies landmarks but also enriches them with rich semantic meaning.

### 2.3 Feature representation

In computer vision, Convolutional Neural Networks (CNNs) have emerged as the predominant method for representing features in visual tasks, owing to their efficient local receptive fields and weight-sharing mechanisms. Although CNNs perform well in many vision tasks, they face challenges with long-range dependencies. To tackle this challenge, Transformer [34] was initially introduced in the NLP field. It adeptly captures global dependencies via its self-attention mechanism and has been demonstrated to excel in processing sequential data. ViT [35] successfully applied Transformer technology for computer vision, achieving notable success and proving its effectiveness in extracting visual features. DETR [36] expanded the use of the Transformer to object detection, enabling end-to-end training and inference. Swin Transformer [37] introduced innovative shift window and cross-window connection mechanisms, exhibiting superior performance in varying visual tasks such as image classification, object detection, and image segmentation. TransFlow [38], a pure Transformer architecture for optical flow estimation, could serve as a flexible baseline for optical flow estimation tasks. These studies highlighted the Transformer’s capabilities in visual tasks and pave the way for new research avenues in vision.

However, the field of unsupervised landmark detection has seen relatively limited research on employing the Transformer architecture for feature representation. In landmark detection tasks, understanding the global interaction among landmarks and their overall spatial distribution is essential for improving landmark detection performance. Therefore, we integrate global self-attention mechanisms from the Transformer architecture into our model for unsupervised landmark detection.

## 3 Methods

In this section, we introduce *SCTN*, an innovative Siamese comparative transformer-based network designed to predict landmark locations within images. Specifically, the overall framework of *SCTN* is elaborated in Section 3.1. Following this, the Siamese contrastive regularizer and the direction-guided Transformer-based image pose encoder are introduced in Section 3.2 and Section 3.3, respectively. Finally, a concise overview of the training objective is provided in Section 3.4.

### 3.1 Overall framework

Given a set of image pairs, source image $x\in R^{3\times H^{\prime }\times W^{\prime }}$ and target image $x^{\prime }\in R^{3\times H^{\prime }\times W^{\prime }}$, each pair has the same appearances but different poses. Our objective is to train an image pose encoder, Φ_*p*_: *x* → *p*, that maps an object contained in *x* to its pose *p*, without relying on any human annotation. Where $p\in R^{K\times H_{O}\times W_{O}}$ represents the landmark heatmaps with *K* channels, and each channel corresponds to one of the landmarks. Hence, we can obtain the estimated coordinates for the *i*-th landmark by calculating the expectation over the *i*-th channel of *p*.

**Fig 2:**
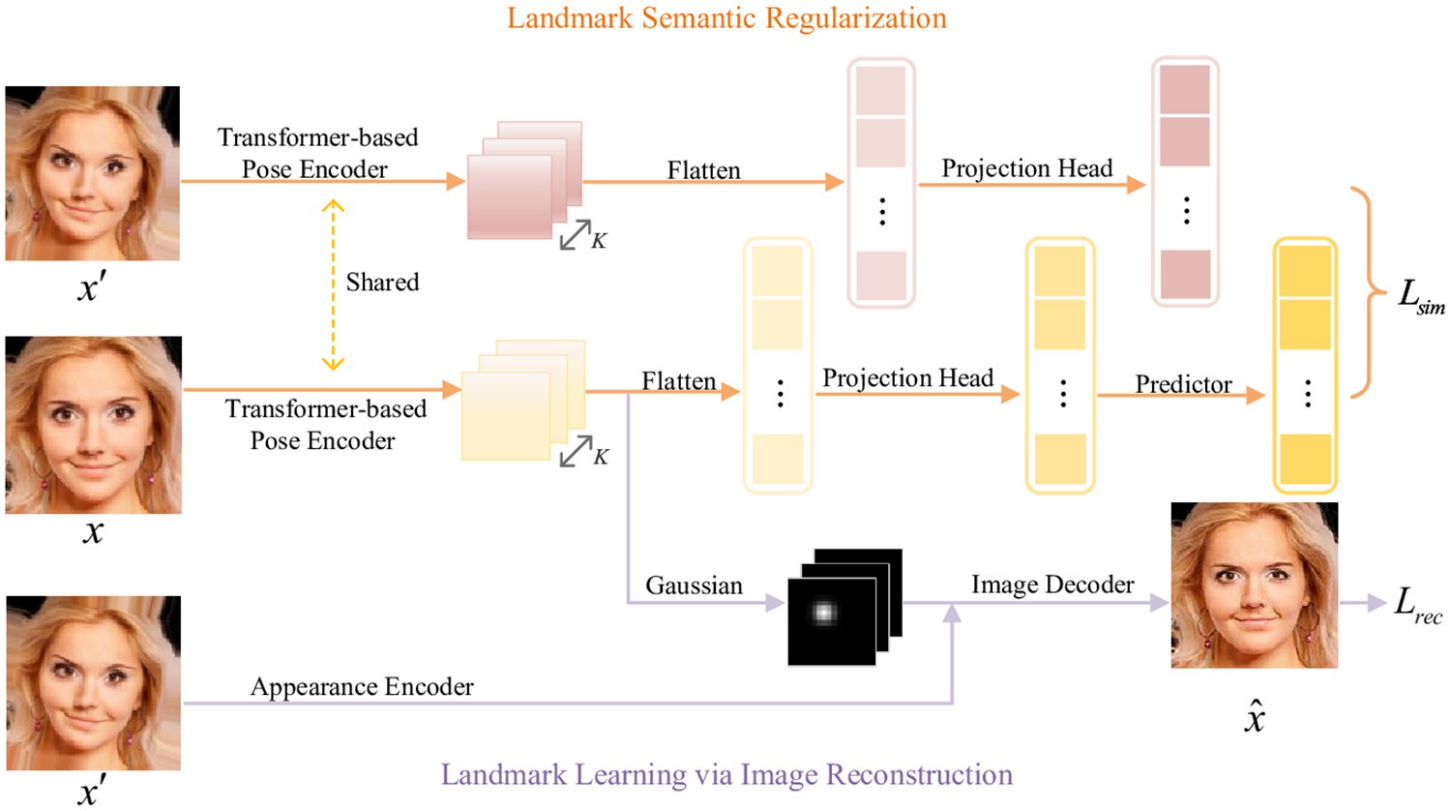
The overall framework of our proposed SCTN. It consists of a semantic regularization process and an image reconstruction stage. Specifically, our method utilizes a shared-weight Siamese pose encoding network to extract landmark feature embeddings from both input image pairs, aiming to bring feature embeddings closer in a high-dimensional semantic space. Furthermore, a direction-guided transformer-based image pose encoder encodes the global positional relationships of landmarks, leading to more precise landmark localization(The human face images in this figure come from the dataset AFLW [39]).

The comprehensive framework of our *SCTN* is illustrated in Fig 2, which mainly contains two parts: landmark semantic regularization and landmark learning via image reconstruction. In the landmark semantic regularization part, the *Siamese contrastive regularizer* draws semantically consistent landmark pairs closer together in the feature space, thereby imposing semantic constraints on the landmarks predicted by the image pose encoder.

In the image reconstruction part, initially, an image appearance encoder Φ_*a*_ processes the deformed version of the source image $x^{\prime }\in R^{3\times H^{\prime }\times W^{\prime }}$ to extract an appearance visual feature map, denoted as *a*. Subsequently, a *direction-guided Transformer-based image pose encoder* Φ_*p*_ which enhances landmark detection capabilities by capturing directional features and establishing long-range dependencies is applied to the source image $x\in R^{3\times H^{\prime }\times W^{\prime }}$ to extract landmark heatmaps, denoted as *p*. Finally, the appearance visual feature map *a* and landmark heatmaps *p* are concatenated, and then fed into the image decoder Ψ, generating the target image $\hat{x}$. The process can be described as follows:

$$\begin{array}{c} \begin{array}{cc} & a=\Phi_{a}\left(x^{\prime }\right),\\ & p=\Phi_{p}\left(x\right),\\ & \hat{x}=\Psi \left(a,p\right) \end{array} \end{array}$$ (1)

To this end, employing the Transforemr-based image pose encoder Φ_*p*_ with semantic regularization can effectively improve the landmark detection performance.

### 3.2 Siamese contrastive regularizer

In our method, target image *x*′ represents an augmented view of the source image *x*, deformed by the thin plate spline transformation. Thus, the geometric positions of object landmarks in *x*′ differ from those in *x*, while the semantics of corresponding landmarks, like the eyes, nose, and mouth, should remain unchanged. Consequently, we introduce a Siamese contrastive regularizer to maintain semantic consistency among counterpart landmark pairs and guarantee that the landmarks identified by the pose encoder hold semantic validity. As illustrated in Fig 2, we incorporate an additional image pose encoder branch that shares the same parameters with the original image pose encoder branch. These two branches form a Siamese structure, facilitating the contrastive learning of landmark semantic features.

First, a pair of images *x* and *x*′ is passed through a shared image pose encoder network, yielding corresponding feature maps in the two branches. These feature maps encode the geometric locations of landmarks. The formulation can be described as follows:

$$\begin{array}{c} \left(p,p^{\prime }\right)=\Phi_{p}\left(x,x^{\prime }\right) \end{array}$$ (2)

where $p\in R^{K\times H_{O}\times W_{O}}$ and $p^{\prime }\in R^{K\times H_{O}\times W_{O}}$ are 2D feature maps with K channels. Subsequently, the 2D landmark feature maps *p* and *p*′ are flattened into 1D embeddings *e* and *e*′. The embeddings *e* and *e*′ are processed via a projection head to produce contrastive embedding pairs (*z*, *z*′). It can be expressed as follows:

$$\begin{array}{c} \left(z,z^{\prime }\right)=G\left(e,e^{\prime }\right) \end{array}$$ (3)

where *G*(⋅) represents the projection head. It comprises three blocks, with each block containing a fully connected layer, batch normalization, and a ReLU layer. Moreover, a predictor, denoted as *H*, transforms the contrastive embeddings of one image and matches it to the other image. Where *H*(⋅) consists of two fully connected layers.

Donating the two output embeddings as *s* = *H*(*z*) and *s*′ = *H*(*z*′). We design an asymmetric architecture between the two branches and minimize the negative cosine similarity between *z* and *s*′, and *z*′ and *s*. The cosine similarity function can be expressed as:

$$\begin{array}{c} D\left(z,s^{\prime }\right)=-\frac{z\cdot s^{\prime }}{{∥z∥}_{2}\cdot {∥s^{\prime }∥}_{2}} \end{array}$$ (4)

Then, we add the stopping gradient operation *stopgrad*(⋅) and define the similarity loss as:

$$\begin{array}{c} L_{sim}=\frac{1}{2}D\left(z,stopgrad\left(s^{\prime }\right)\right)+\frac{1}{2}D\left(z^{\prime },stopgrad\left(s\right)\right) \end{array}$$ (5)

Specifically, the image pose encoder processing image *x*′ receives no gradient from *s*′, yet it receives a gradient from *z*. Conversely, the image pose encoder processing image *x* does not receive a gradient from *s*, but it does from *z*′. By minimizing *L*_*sim*_, the image pose encoder concentrates on preserving the semantic validity of individual landmarks and ensuring the semantic consistency of corresponding landmark pairs.

### 3.3 Direction-guided transformer-based image pose encoder

**Fig 3:**
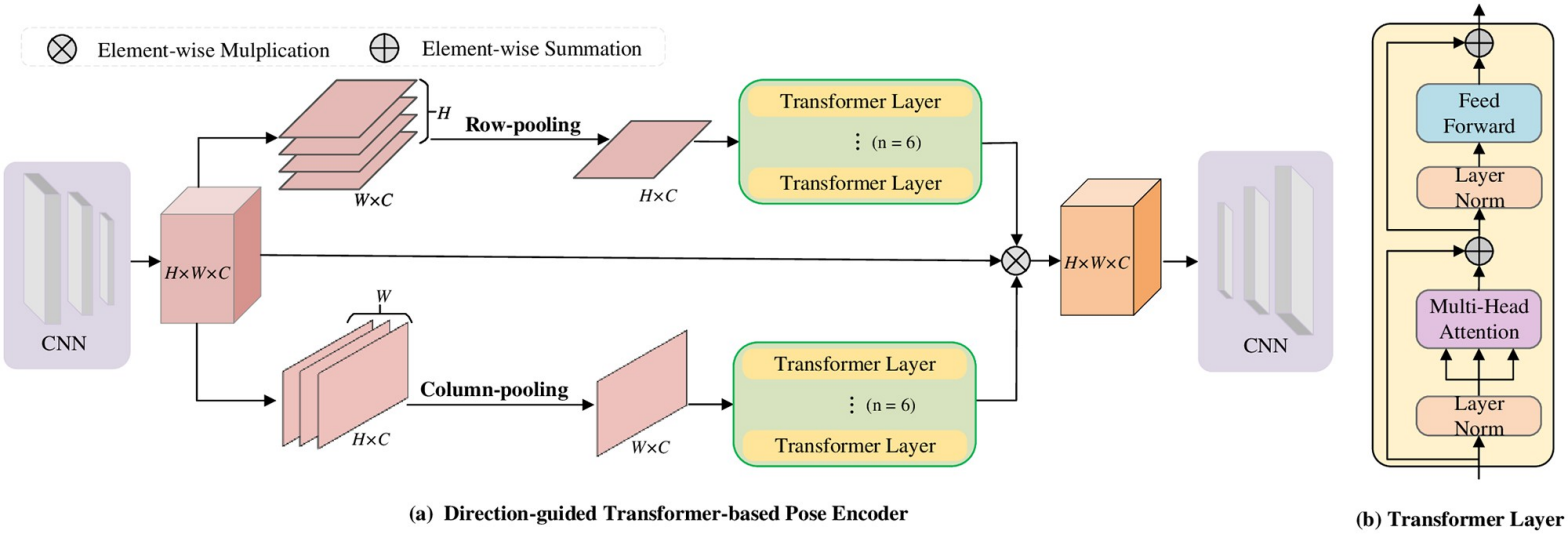
The structure of direction-guided transformer-based image pose encoder. The input feature map is divided into height and width directions, both of which are input into the respective Transformer layers to acquire global-aware information.

Transformer encoder overcomes the convolutional layer’s local receptive field limitations, capturing global features to improve feature representation. Furthermore, global information is crucial for the image pose encoder to understand the overall landmark relationship. To this end, We propose a direction-guided Transformer-based image pose encoder. The detailed structure is depicted in Fig 3. The image pose encoder integrates an Unet architecture [40] with a direction-guided Transformer to address the computational complexity encountered by standard Transformer encoder [36] in processing image sequences. The direction-guided Transformer compresses feature maps into 1D sequences along specific directions, effectively reducing computational complexity while preserving global spatial information. The Unet architecture features a symmetric upsampling-downsampling network with skip connections for multi-scale feature fusion. Selected as the backbone, the Unet downsampler processes the input image $x\in R^{3\times H^{\prime }\times W^{\prime }}$, transforming *x* into feature maps $f\in R^{C\times H\times W}$. Then, we use spatial adaptive pooling in the height and width directions to obtain two direction-aware feature sequences, which can be expressed as follows:

$$\begin{array}{cc} & f_{h}=\frac{1}{W}\sum_{1\leq i\leq W}f\left(h,i\right) \end{array}$$ (6)

$$\begin{array}{cc} & f_{w}=\frac{1}{H}\sum_{1\leq j\leq H}f\left(j,w\right) \end{array}$$ (7)

where $f_{h}\in R^{C\times H}$ and $f_{w}\in R^{C\times W}$, *H* and *W* are the length of the sequence, and *C* is the dimension of the feature embeddings. Rather than flattening *f* and applying self-attention, we input the compressed *f*_*h*_ and *f*_*w*_ into the Transformer layer, respectively.

The multi-head self-attention mechanism plays a pivotal role in sequence processing within the Transformer layer. Given a *D*-dimensional input sequence *X* of length *N*, the output global self-attention matrix *Y* is calculated as follows:

$$\begin{array}{cc} & Q=W_{Q}X,\;K=W_{K}X,\;V=W_{V}X \end{array}$$ (8)

$$\begin{array}{cc} & A=softmax(\frac{QK^{T}}{\sqrt{D}})V \end{array}$$ (9)

*W*_*Q*_, *W*_*K*_, and *W*_*V*_ denote *D* × *D* parameter matrices, mapping sequence *X* to keys, queries, and values, respectively. The output sequence *A* comprises items derived from linearly combining values *V*. We employ two separate Transformer Encoders for *f*_*h*_ and *f*_*w*_ to individually derive enhanced embeddings, denoted as *z*_*h*_ and *z*_*w*_.

$$\begin{array}{cc} & z_{h}=\sigma \left(T_{h}\left(f_{h}\right)\right) \end{array}$$ (10)

$$\begin{array}{cc} & z_{w}=\sigma \left(T_{w}\left(f_{w}\right)\right) \end{array}$$ (11)

Here, **T**_*h*_ and **T**_*w*_ denote the Transformer Encoder for *f*_*h*_ and *f*_*w*_, while *σ*(⋅) represents the sigmoid activation function. We extend *z*_*h*_ and *z*_*w*_ along the width and height directions, treating them as attention maps. The global-aware output in the *c*-th channel at position (*x*, *y*) can be calculated using the following formula:

$$\begin{array}{c} q\left(x,y\right)=z_{h}\left(x\right)\times z_{w}\left(y\right)\times f\left(x,y\right) \end{array}$$ (12)

Finally, the global-aware feature map *q* is upsampled by the Unet upsampler to generate the final heatmaps $p\in R^{K\times H_{O}\times W_{O}}$.

In our model, we stack 6 encoder layers for obtaining the complete representations $q\in R^{C\times H\times W}$. Applying the self-attention mechanism to high-resolution feature maps tends to slow down inference speed, leading to its rare application in landmark detection in previous studies. However, our innovative strategy considerably lowers computational complexity and maintains detection accuracy.

### 3.4 Training objective

Unsupervised landmark detection methods based on image reconstruction adopt reconstruction loss to drive the training of the entire autoencoder. The reconstruction loss can be described as:

$$\begin{array}{c} L_{rec}=\sum_{l}\alpha_{l}{∥\Gamma_{l}\left(x\right)-\Gamma_{l}\left(\hat{x}\right)∥}_{2}^{2} \end{array}$$ (13)

Where *Γ*_*l*_(⋅) represents the feature map of the *l* − *th* layer in the feature extraction network, *α*_*l*_ is the weight of each layer, and ‖⋅‖_2_ represents the *ℓ*_2_-norm. Reconstruction loss evaluates the discrepancies between the source image *x* and the reconstructed image $\hat{x}$ across the feature maps at each layer. By minimizing the reconstruction loss, the network focuses on reconstructing image $\hat{x}$ as close as possible to the source image *x*. The full loss of our model comprises both reconstruction loss and similarity loss proposed by the Siamese contrastive regularizer:

$$\begin{array}{c} L_{all}=\lambda L_{rec}+\mu L_{sim} \end{array}$$ (14)

where λ and *μ* are loss-balancing factors.

It is worth noting that this loss function involves no prior on desired landmarks, and puts no restrictions on the used image pairs. Our model is trained end-to-end unsupervised with an overall loss of *L*_*all*_.

## 4 Experiments and results

In this section, we initially offer detailed insights into the datasets and evaluation indicator in Section 4.1 and Section 4.2, respectively. Then, the training details are illustrated in Section 4.3. Next, comparisons with preceding landmark detection methods and visualizations are detailed in Sections 4.4. Lastly, Section 4.5 discusses the ablation study results.

### 4.1 Datasets

We evaluated the performance of our proposed method on the human faces and cat heads using the CelebA [41], MAFL [7], AFLW [39], and Cat Heads [42] datasets. All images are standardized to a resolution of 128 × 128 pixels.

- The CelebA dataset comprises more than 200k training images of celebrities’ faces.
- The AFLW and MAFL dataset contains 10,122/2,991 and 18,997/1,000 training/testing images respectively.
- Regarding the Cat Heads dataset, nearly 9,000 images are available and contains 7,747/1,257 training/testing images.

### 4.2 Evaluation indicator

Following the previous methods [13, 43], our model is initially trained on the CelebA dataset, excluding the MAFL subset. Subsequently, it undergoes fine-tuning and testing on the MAFL, AFLW, and Cat Heads datasets. In the training phase, the model is configured to predict K = 10 landmarks. In the testing phase, these landmarks undergo linear regression to output 5 points for human faces and 9 points for cat heads. This process facilitates a direct comparison with the labelled points, in terms of both quantity and position. The error between the regressed landmarks and the ground truth points is calculated using the point-to-point mean squared error (MSE), and normalized by the inter-ocular distance (IOD). The evaluation metric can be defined as follows:

$$\begin{array}{c} MSE=\frac{1}{N}\sum_{i=1}^{N}\frac{\sqrt{{\left(c_{x}^{i}-{\hat{c}}_{x}^{i}\right)}^{2}+{\left(c_{y}^{i}-{\hat{c}}_{y}^{i}\right)}^{2}}}{IOD} \end{array}$$ (15)

where *N* denotes the number of regressed landmarks. $\left(c_{x}^{i},c_{y}^{i}\right)$ represents the *i* − *th* coordinate of predicted point, and $\left({\hat{c}}_{x}^{i},{\hat{c}}_{y}^{i}\right)$ represents the *i* − *th* coordinate of labelled point.

### 4.3 Training details

In the training phase, the model weights are initialized using Xavier initialization. The VGG-19 [44] is adopted as the feature extraction network. As specified in Eq (13), *l* = {*relu*1_−_2, *relu*2_−_2, *relu*3_−_3, *relu*4_−_3} represents the selected layers, with weights $\alpha_{l}=\{\frac{1}{32},\frac{1}{16},\frac{1}{8},\frac{1}{4}\}$, respectively. The Adam optimizer with an initial learning rate of 0.00001 is used to train our model, and the total training epochs is 150. All experiments are conducted on 8 NVIDIA RTX 3090 GPUs with a batch size of 32.

### 4.4 Comparison with state-of-the-arts

**Fig 4:**
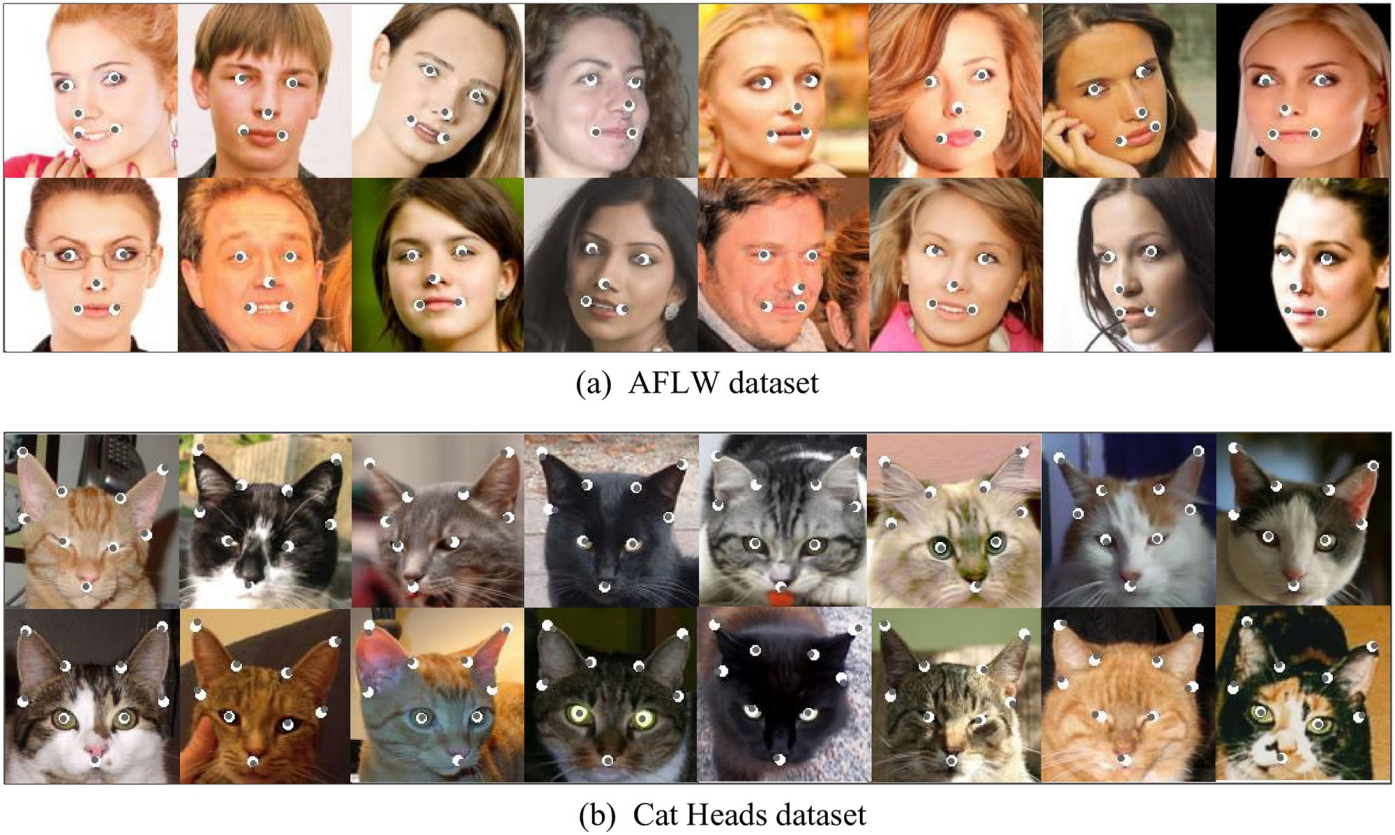
Visualization results of our method on the AFLW [39] and Cat Heads [42] datasets. The white solid circles represent labelled points and the grey solid circles represent points predicted by our Transformer-based image pose encoder.

To validate the efficacy of our proposed method, we compare it with existing advanced models on the test sets of the MAFL, AFLW, and Cat Heads. The quantitative results are presented in Tables 1–3, with the comparative analysis based on the mean squared error (MSE) metric. The MSE values for our model are reported as 3.76% on the MAFL dataset, 6.96% on the AFLW dataset, and 13.25% on the Cat Heads dataset. This performance surpasses all other unsupervised methods and even outperforms most supervised learning methods. Moreover, we map the output of the image pose encoder into landmarks via regression operations. The visualization of these results on the AFLW and Cat Heads datasets is illustrated in Fig 4. The white solid circles represent labelled points, while the grey solid circles represent points predicted by our image pose encoder. It can be found that our model shows good performance in these three datasets. Despite challenges like role variations and appearance differences, the landmarks derived from our model precisely align with targeted areas, closely matching the locations of the labelled points.

**Table 1:** Comparison of our model with others on MAFL dataset.

Model	Unsupervised Learning	MSE
CFAN [17]	✘	15.84
Cas CNN [16]	✘	9.73
TCNDCN [8]	✘	7.95
MTCNN [7]	✘	5.39
Sahasrabudhe [45]	✔	6.01
Thewlis [18]	✔	5.83
Shu [13]	✔	5.45
Rahul [46]	✔	4.59
Mails [20]	✔	4.12
He [47]	✔	3.94
Ours	✔	**3.76**

**Table 2:** Comparison of our model with others on AFLW dataset.

Model	Unsupervised Learning	MSE
RCPR [9]	✘	11.60
CFAN [17]	✘	10.94
Cas CNN [16]	✘	8.97
TCNDCN [8]	✘	7.65
MTCNN [7]	✘	6.90
Thewlis [18]	✔	8.80
Mails [20]	✔	7.37
Zhang [43]	✔	7.01
Sanchez [12]	✔	6.99
Ours	✔	**6.96**

**Table 3:** Comparison of our model with others on Cat Heads dataset.

Model	Unsupervised Learning	MSE
Thewlis [11]	✔	26.76
Zhang [43]	✔	16.35
Rahul [46]	✔	14.74
Ours	✔	**13.25**

### 4.5 Ablation study

In this subsection, we examine the impact of three key factors to verify the effectiveness of our method *SCTN*: (1) effectiveness of components; (2) Hyperparameters λ and *μ*; and (3) model parameters.

#### 4.5.1 Effectiveness of components

The Siamese network for landmark extraction guarantees both the semantic validity of individual landmarks and their semantic consistency across landmark pairs. The internal self-attention mechanism of the Transformer structure constructs global feature information, thus enhancing the model’s capability to represent accurate landmarks. In order to confirm the effectiveness of the proposed components, ablation experiments are conducted on the MAFL test set. The results are presented in Table 4.

**Table 4:** Ablation studies of the proposed components on the MAFL dataset.

Unet	Siamese contrastive regularizer	Direction-guided Transformer	MSE
✔			5.47
✔	✔		4.46
✔		✔	4.67
✔	✔	✔	3.76

Without the configuration of the Siamese contrastive regularizer and direction-guided Transformer, the image pose encoder reaches a landmark error of 5.47. Equipping the image pose encoder with the Siamese contrastive regularizer results in a landmark error of 4.46, reducing the MSE value by 1.01. Additionally, incorporating the direction-guided Transformer into the image pose encoder yields a landmark error of 4.67, lowering the MSE value by 0.8. These findings validate the effectiveness of the proposed components. Furthermore, when the Siamese contrastive regularizer and direction-guided Transformer are simultaneously implemented, the landmark error reduces to 3.76, underscoring the superior performance of our model.

#### 4.5.2 Hyperparameters

Based on Eq (14), the loss of the whole algorithm is composed of similarity loss and reconstruction loss. Theoretically, these two losses impact model performance in different magnitudes. Directly summing these two losses without adjusting for their distinct effects is not advisable. To find the optimal balance between the two losses, we conducted experiments on loss function weight. Various loss weights were applied in the experimental settings, and the MSE was computed. The results are presented in Table 5. It can be observed that the model achieves an optimal MSE value of 3.76 when λ = 0.3 and *μ* = 0.7. Therefore, the final loss formula in our experiment is loss = 0.3 × reconstruction loss + 0.7 × similarity loss.

**Table 5:** Results of different loss function weight.

Reconstruction loss	Similarity loss	MSE
0.1	0.9	3.99
0.2	0.8	4.03
0.3	0.7	3.86
0.4	0.6	3.97
0.5	0.5	4.00
0.6	0.4	4.04
0.7	0.3	**3.76**
0.8	0.2	3.90
0.9	0.1	3.83

#### 4.5.3 Model parameters

The experiment results of our proposed model demonstrate that the Transformer architecture utilizes a multi-head self-attention mechanism to efficiently encode landmarks. The direct application of a standard Transformer [34] will increase the model’s complexity. Hence, we introduce a novel direction-guided Transformer, which reduces learnable parameters by compressing feature maps while retaining global modeling capabilities. However, it is worth discussing whether the proposed direction-guided Transformer, despite lowering computational complexity, also leads to a reduction in model performance when compared to the standard Transformer. The comparison results are shown in Table 6. It is observed that integrating the Unet with the standard Transformer enhances the landmark extraction capability of the image pose encoder, but increases the model’s parameters to a maximum of 34.7M and the floating point operations (FLOPs) of the model to 47.3G. Conversely, when the Unet is equipped with the direction-guided Transformer, there is a slight reduction in parameters to 31.3M and the FLOPs to 43.6G, while achieving an optimal MSE value of 4.67.

**Table 6:** Impact of model components on model efficiency and performance.

Model	Params	FLOPs	MSE
Unet	20.6M	42.4G	5.47
Unet+standard Transformer	34.7M	47.3G	5.11
Unet+direction-guided Transformer	31.3M	43.6G	**4.67**

Additionally, we compare the computational efficiency of our proposed method with the other two methods in Table 7. In these three mentioned methods, our proposed model demonstrates the best performance. Our proposed method has a parameter size of 31.3M, slightly higher than the other methods, it achieves lower FLOPs of 43.6G compared to [47] at 49.7G, while maintaining the lowest MSE of 3.76. We found that the small number of parameters and high FLOPs in method [20, 47] are due to using larger feature maps. These results highlight that our model achieves the best balance between accuracy and computational efficiency. It provides higher precision with reduced complexity, making it more suitable for applications where both accuracy and resource efficiency are critical.

**Table 7:** Comparison of model computational efficiency and performance.

Model	Params	FLOPs	MSE
Mails [20]	8.4M	41.6G	4.12
He [47]	25.7M	49.7G	3.94
Ours	31.3M	43.6G	3.76

## 5 Conclusion

In this paper, we propose a novel unsupervised landmark learning method for achieving precise landmark representation. This approach employs a Siamese contrastive regularizer, leveraging a Siamese network from contrastive learning to bolster the semantic consistency of detected landmarks. At the same time, a lightweight direction-guided Transformer is proposed to augment the image pose encoder’s global perception capabilities for landmark detection tasks. Experimental results indicate that our method excels in preserving the semantic consistency of landmarks and improving landmark detection accuracy.

## References

[1] Newell A, Yang K, Deng J. Stacked Hourglass Networks for Human Pose Estimation. In: Proceedings of the European conference on computer vision (ECCV); 2016. p. 483–499.

[2] Cao Z, Simon T, Wei SE, Sheikh Y. Realtime Multi-person 2D Pose Estimation Using Part Affinity Fields. In: IEEE Conference on Computer Vision and Pattern Recognition (CVPR); 2017. p. 1302–1310. 10.1109/TPAMI.2019.2929257 31331883

[3] Du Y, Wang W, Wang L. Hierarchical recurrent neural network for skeleton based action recognition. In: IEEE Conference on Computer Vision and Pattern Recognition (CVPR); 2015. p. 1110–1118.

[4] Yan S, Xiong Y, Lin D. Spatial temporal graph convolutional networks for skeleton-based action recognition. In: Proceedings of the AAAI conference on artificial intelligence. vol. 32; 2018.

[5] Siarohin A, Lathuiliere S, Tulyakov S, Ricci E, Sebe N. Animating Arbitrary Objects via Deep Motion Transfer. In: IEEE Conference on Computer Vision and Pattern Recognition (CVPR); 2019. p. 2372–2381.

[6] Zhang J, Mao H. WKNN indoor positioning method based on spatial feature partition and basketball motion capture. Alexandria engineering journal. 2022;61(1):125–134. doi: 10.1016/j.aej.2021.04.078

[7] Zhang Z, Luo P, Loy CC, Tang X. Facial Landmark Detection by Deep Multi-task Learning. In: Proceedings of the European conference on computer vision (ECCV); 2014. p. 94–108.

[8] Zhang Z, Luo P, Loy CC, Tang X. Learning deep representation for face alignment with auxiliary attributes. IEEE transactions on pattern analysis and machine intelligence. 2015;38(5):918–930. doi: 10.1109/TPAMI.2015.2469286 27046839

[9] Burgos-Artizzu XP, Perona P, Dollár P. Robust face landmark estimation under occlusion. In: Proceedings of the IEEE international conference on computer vision(ICCV); 2013. p. 1513–1520.

[10] Zhang T, Zhang Z, Zhu X, Chen B, Li J, Zhong Y. Aircraft engine danger areas incursion detection using keypoint detection and IoT. Alexandria Engineering Journal. 2024;93:7–21. doi: 10.1016/j.aej.2024.03.003

[11] Thewlis J, Bilen H, Vedaldi A. Unsupervised learning of object landmarks by factorized spatial embeddings. In: Proceedings of the IEEE international conference on computer vision(ICCV); 2017. p. 5916–5925.

[12] Sanchez E, Tzimiropoulos G. Object landmark discovery through unsupervised adaptation. Advances in Neural Information Processing Systems. 2019;32.

[13] Shu Z, Sahasrabudhe M, Alp Güler R, Samaras D, Paragios N, Kokkinos I. Deforming Autoencoders: Unsupervised Disentangling of Shape and Appearance. In: Proceedings of the IEEE international conference on computer vision(ICCV); 2018. p. 664–680.

[14] Lorenz D, Bereska L, Milbich T, Ommer B. Unsupervised Part-Based Disentangling of Object Shape and Appearance. In: IEEE Conference on Computer Vision and Pattern Recognition (CVPR); 2019. p. 10947–10956.

[15] Mallis D, Sanchez E, Bell M, Tzimiropoulos G. From Keypoints to Object Landmarks via Self-Training Correspondence: A novel approach to Unsupervised Landmark Discovery. IEEE Transactions on Pattern Analysis and Machine Intelligence. 2023; p. 1–15. doi: 10.1109/TPAMI.2023.3234212 37018262

[16] Sun Y, Wang X, Tang X. Deep convolutional network cascade for facial point detection. In: Proceedings of the IEEE conference on computer vision and pattern recognition(CVPR); 2013. p. 3476–3483.

[17] Zhang J, Shan S, Kan M, Chen X. Coarse-to-Fine Auto-Encoder Networks (CFAN) for Real-Time Face Alignment. In: Proceedings of the IEEE international conference on computer vision(ICCV); 2014. p. 1–16.

[18] Thewlis J, Bilen H, Vedaldi A. Unsupervised object learning from dense equivariant image labelling. Advances in Neural Information Processing Systems. 2017;8.

[19] Thewlis J, Albanie S, Bilen H, Vedaldi A. Unsupervised learning of landmarks by descriptor vector exchange. In: Proceedings of the IEEE international conference on computer vision(ICCV); 2019. p. 6360–6370.

[20] Mallis D, Sanchez E, Bell M, Tzimiropoulos G. Unsupervised learning of object landmarks via self-training correspondence. Advances in Neural Information Processing Systems. 2020;33:4709–4720.

[21] Wang Y, Lu Y, Xie Z, Lu G. Deep unsupervised 3d sfm face reconstruction based on massive landmark bundle adjustment. In: Proceedings of the 29th ACM International Conference on Multimedia. vol. 33; 2021. p. 1350–1358.

[22] Wan J, Liu J, Zhou J, Lai Z, Shen L, Sun H, et al. Precise facial landmark detection by reference heatmap transformer. IEEE Transactions on Image Processing. 2023;32:1966–1977. doi: 10.1109/TIP.2023.3261749 37030695

[23] Jakab T, Gupta A, Bilen H, Vedaldi A. Unsupervised Learning of Object Landmarks through Conditional Image Generation. Advances in neural information processing systems. 2018;31.

[24] Li W, Liao H, Miao S, Lu L, Luo J. Unsupervised Learning of Facial Landmarks based on Inter-Intra Subject Consistencies. In: 2020 25th International Conference on Pattern Recognition (ICPR); 2021.

[25] Wu T, Fan W, Li S, Li Q, Zhang J, Li M. Unsupervised detection of contrast enhanced highlight landmarks. IET Computer Vision. 2023;.

[26] Wu Z, Xiong Y, Yu SX, Lin D. Unsupervised Feature Learning via Non-parametric Instance Discrimination. In: Proceedings of the IEEE conference on computer vision and pattern recognition(CVPR); 2018. p. 3733–3742.

[27] Bromley J, Guyon I, LeCun Y, Säckinger E, Shah R. Signature verification using a “siamese” time delay neural network. Advances in neural information processing systems. 1993;6.

[28] Grill JB, Strub F, Altché F, Tallec C, Richemond P, Buchatskaya E, et al. Bootstrap your own latent-a new approach to self-supervised learning. Advances in neural information processing systems. 2020;33:21271–21284.

[29] Caron M, Misra I, Mairal J, Goyal P, Bojanowski P, Joulin A. Unsupervised learning of visual features by contrasting cluster assignments. Advances in neural information processing systems. 2020;33:9912–9924.

[30] He K, Fan H, Wu Y, Xie S, Girshick R. Momentum Contrast for Unsupervised Visual Representation Learning. In: Proceedings of the IEEE conference on computer vision and pattern recognition(CVPR); 2020. p. 9726–9735.

[31] Pan T, Song Y, Yang T, Jiang W, Liu W. Videomoco: Contrastive video representation learning with temporally adversarial examples. In: Proceedings of the IEEE conference on computer vision and pattern recognition(CVPR); 2021. p. 11205–11214.

[32] Chen T, Kornblith S, Norouzi M, Hinton GE. A Simple Framework for Contrastive Learning of Visual Representations. International Conference on Machine Learning. 2020; p. 1597–1607.

[33] Chen X, He K. Exploring Simple Siamese Representation Learning. In: Proceedings of the IEEE conference on computer vision and pattern recognition(CVPR); 2021. p. 15745–15753.

[34] Vaswani A, Shazeer N, Parmar N, Uszkoreit J, Jones L, Gomez AN, et al. Attention is all you need. Advances in neural information processing systems. 2017;30.

[35] Dosovitskiy A, Beyer L, Kolesnikov A, Weissenborn D, Zhai X, Unterthiner T, et al. An Image is Worth 16x16 Words: Transformers for Image Recognition at Scale. International Conference on Learning Representations. 2020;.

[36] Carion N, Massa F, Synnaeve G, Usunier N, Kirillov A, Zagoruyko S. End-to-End Object Detection with Transformers. In: Proceedings of the European conference on computer vision (ECCV); 2020. p. 213–229.

[37] Liu Z, Lin Y, Cao Y, Hu H, Wei Y, Zhang Z, et al. Swin Transformer: Hierarchical Vision Transformer using Shifted Windows. In: Proceedings of the IEEE international conference on computer vision(ICCV); 2021. p. 9992–10002.

[38] Lu Y, Wang Q, Ma S, Geng T, Chen YV, Chen H, et al. Transflow: Transformer as flow learner. In: Proceedings of the IEEE conference on computer vision and pattern recognition(CVPR); 2023. p. 18063–18073.

[39] Koestinger M, Wohlhart P, Roth PM, Bischof H. Annotated facial landmarks in the wild: A large-scale, real-world database for facial landmark localization. In: 2011 IEEE international conference on computer vision workshops (ICCV workshops); 2011. p. 2144–2151.

[40] Ronneberger O, Fischer P, Brox T. U-net: Convolutional networks for biomedical image segmentation. In: Medical Image Computing and Computer-Assisted Intervention; 2015. p. 234–241.

[41] Liu Z, Luo P, Wang X, Tang X. Deep learning face attributes in the wild. In: Proceedings of the IEEE international conference on computer vision(CVPR); 2015. p. 3730–3738.

[42] Zhang W, Sun J, Tang X. Cat head detection-how to effectively exploit shape and texture features. In: Proceedings of the IEEE conference on computer vision and pattern recognition(CVPR); 2008. p. 802–816.

[43] Zhang Y, Guo Y, Jin Y, Luo Y, He Z, Lee H. Unsupervised Discovery of Object Landmarks as Structural Representations. In: Proceedings of the IEEE conference on computer vision and pattern recognition(CVPR); 2018. p. 2694–2703.

[44] Simonyan K, Zisserman A. Very deep convolutional networks for large-scale image recognition. arXiv preprint arXiv:14091556. 2014;.

[45] Sahasrabudhe M, Shu Z, Bartrum E, Alp Guler R, Samaras D, Kokkinos I. Lifting autoencoders: Unsupervised learning of a fully-disentangled 3d morphable model using deep non-rigid structure from motion. In: Proceedings of the IEEE/CVF International Conference on Computer Vision Workshops; 2019. p. 0–0.

[46] Rahaman R, Ghosh A, Thiery AH. Pretrained equivariant features improve unsupervised landmark discovery. In: 2022 26th International Conference on Pattern Recognition (ICPR); 2022. p. 4434–4440.

[47] He X, Wandt B, Rhodin H. Autolink: Self-supervised learning of human skeletons and object outlines by linking keypoints. Advances in Neural Information Processing Systems. 2022;35:36123–36141.

